# Combination of genetically diverse *Pseudomonas* phages enhances the cocktail efficiency against bacteria

**DOI:** 10.1038/s41598-023-36034-2

**Published:** 2023-06-01

**Authors:** Ampapan Naknaen, Thanadon Samernate, Wichanan Wannasrichan, Komwit Surachat, Poochit Nonejuie, Vorrapon Chaikeeratisak

**Affiliations:** 1 Department of Biochemistry, Faculty of Science, Chulalongkorn University, Bangkok, Thailand; 2 Institute of Molecular Biosciences, Mahidol University, Nakhon Pathom, Thailand; 3 Department of Biomedical Sciences and Biomedical Engineering, Faculty of Medicine, Prince of Songkla University, Songkhla, Thailand; 4 Translational Medicine Research Center, Faculty of Medicine, Prince of Songkla University, Songkhla, Thailand; 5 Cell and Biomolecular Imaging Research Unit (CBIRU), Department of Biochemistry, Faculty of Science, Chulalongkorn University, Bangkok, Thailand

**Keywords:** Microbiology, Bacteriophages, Phage biology

## Abstract

Phage treatment has been used as an alternative to antibiotics since the early 1900s. However, bacteria may acquire phage resistance quickly, limiting the use of phage treatment. The combination of genetically diverse phages displaying distinct replication machinery in phage cocktails has therefore become a novel strategy to improve therapeutic outcomes. Here, we isolated and studied lytic phages (SPA01 and SPA05) that infect a wide range of clinical *Pseudomonas aeruginosa* isolates. These relatively small myophages have around 93 kbp genomes with no undesirable genes, have a 30-min latent period, and reproduce a relatively high number of progenies, ranging from 218 to 240 PFU per infected cell. Even though both phages lyse their hosts within 4 h, phage-resistant bacteria emerge during the treatment. Considering SPA01-resistant bacteria cross-resist phage SPA05 and vice versa, combining SPA01 and SPA05 for a cocktail would be ineffective. According to the decreased adsorption rate of the phages in the resistant isolates, one of the anti-phage mechanisms may occur through modification of phage receptors on the target cells. All resistant isolates, however, are susceptible to nucleus-forming jumbophages (PhiKZ and PhiPA3), which are genetically distinct from phages SPA01 and SPA05, suggesting that the jumbophages recognize a different receptor during phage entry. The combination of these phages with the jumbophage PhiKZ outperforms other tested combinations in terms of bactericidal activity and effectively suppresses the emergence of phage resistance. This finding reveals the effectiveness of the diverse phage-composed cocktail for reducing bacterial growth and prolonging the evolution of phage resistance.

## Introduction

Multidrug-resistant pathogens are a major public health concern that are expected to kill 10 million people worldwide each year by 2050^1^. This is, in part, due to the lack of novel classes of antibiotics that are potentially active against WHO critical threat bacteria in recent years^2^. Thus, strategies for using novel therapeutic approaches must be urgently developed to tackle the emerging multidrug-resistant bacteria. Among all other strategies, the use of prokaryotic viruses or bacteriophages as a therapeutic agent is now becoming one of the most promising approaches, as documented in various clinical studies^3–5^ and recently successful phage therapy^6–10^. Unlike conventional antibiotics, which have chemical identities to define their properties and mechanisms of action (MOA), phages have intricated biological identities that they exhibit during the infection cycle to define their antibacterial properties, such as specificity to host cells and mechanisms of hijacking. During infection, phages specifically bind to receptors on the bacterial cell surface, inject their genome, self-replicate by hijacking the fundamental host cell machinery, and lyse the host bacteria to release viral progeny at the end of the lytic cycle^11–13^. Knowledge regarding each infection step is therefore crucial in understanding how phage exerts its antibacterial activities, and studies that reveal these intricate biological properties are thus important for developing an effective phage therapy.

Multiple phages, also called phage cocktails, are highly preferable in phage therapy for better efficacy, and they have been employed to treat a number of patients in several countries, with the treatments coming out successful^3–5^. Through distinct replication mechanisms of the phages in the cocktail, such as adsorption to different receptors^14,15^ and hijacking different molecular targets in bacterial cells^16–20^, phage cocktails usually broaden the host spectrum and, in some cases, prevent or reduce the evolution of phage-resistant bacteria. A previous study investigating the in vitro testing of 827 unique phage combinations of 1–12 phages against *Pseudomonas aeruginosa* demonstrated that maximizing phage functional diversity in phage cocktails is an effective way to design an efficient phage cocktail and to decrease the phage richness in the cocktail^21^. This is because functionally diverse phages tend to target or interfere with different host molecular targets, and mutations at these targets in bacteria rarely occur simultaneously, making phage resistance to the cocktail very unlikely.

One of the most common opportunistic bacteria is *P. aeruginosa*, as it causes bloodstream infections, pneumonia, urinary tract infections, and surgical site infections, which are particularly complicated diseases in patients with compromised host defense^22^. Furthermore, treatments of *P. aeruginosa* infections are extremely difficult because of its rapid intrinsic mutations and adaptation to resist antibiotics^23^. Therefore, application of multiple phages in combinations for *P. aeruginosa* treatment has been attempted, and it has been widely proven promising in both increasing effectiveness and suppressing resistance compared to the use of a single phage^24,25^. Here, we first isolated and characterized the biological properties of two virulent *Pseudomonas* phages: SPA01 and SPA05, for therapeutic use. We then investigated whether they were an appropriate combination for a phage cocktail and what possible anti-phage mechanisms in bacteria would emerge as a result of their use. Since these phages are closely related, we further examined the therapeutic potential of combining them with other distantly related *Pseudomonas* phages: nucleus-forming jumbophages. Through the combinations of the phages and the nucleus-forming jumbophages, we demonstrated efficient cocktail formulas that better suppress bacterial regrowth and prolong the phage resistance.

## Results and discussion

### Phage morphological and biological properties of phages SPA01 and SPA05

**Figure 1:**
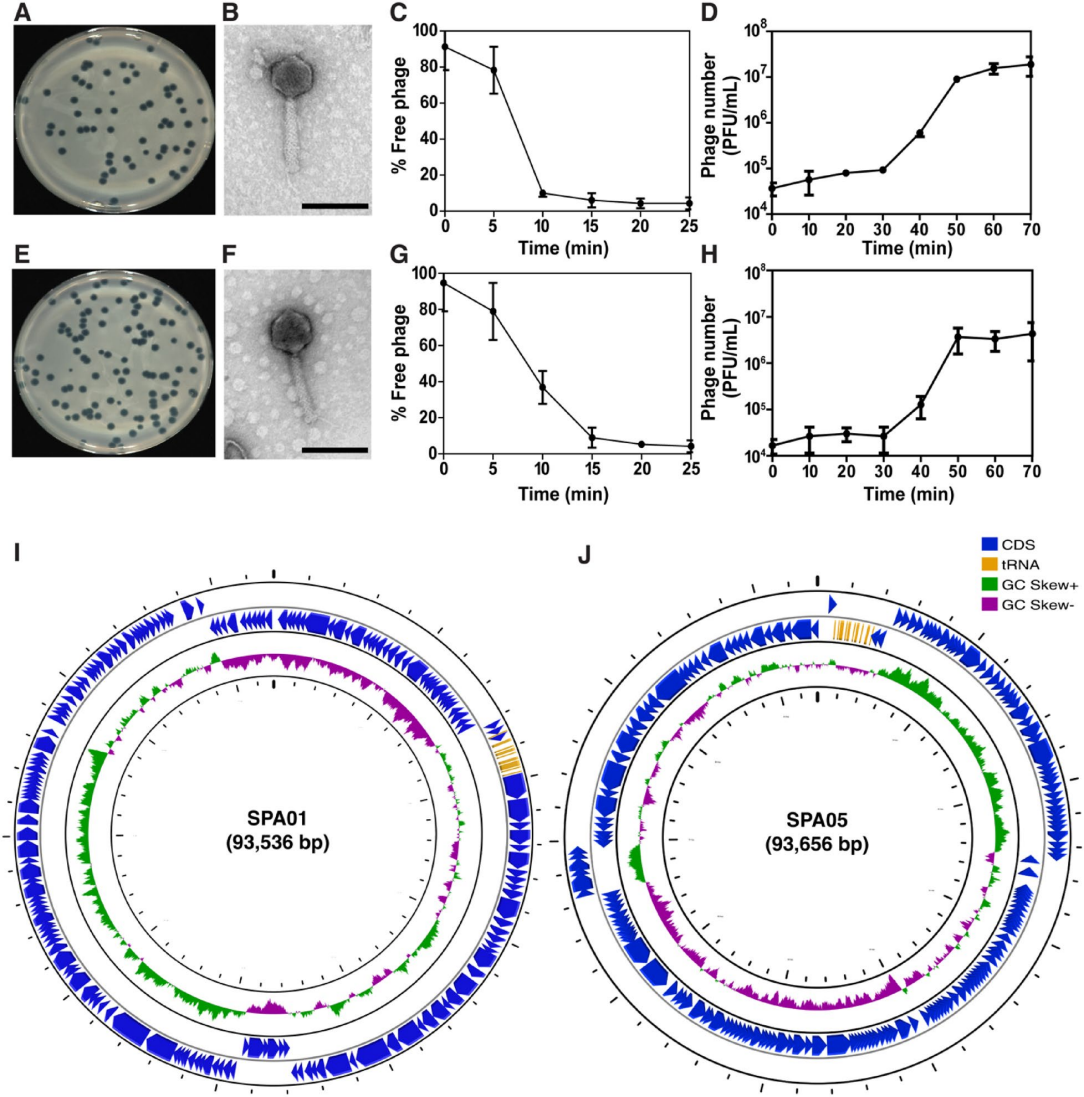
Morphological, biological, and genomic characteristics of phages SPA01 and SPA05. Plaque morphology of phages SPA01 (**A**) and SPA05 (**E**). Transmission electron micrographs of phages SPA01 (**B**) and SPA05 (**F**). Scale bar equals to 100 nm. Adsorption assays within 25 min of phages SPA01 (**C**) and SPA05 (**G**) with *P. aeruginosa* strain PAO1. One-step growth curve of phages SPA01 (**D**) and SPA05 (**H**) in *P. aeruginosa* strain PAO1 during a window of 70 min. Schematic whole genome maps of phages SPA01 (**I**) and SPA05 (**J**). The innermost circles colored in green and purple indicate the positive and negative GC skew, respectively. The open reading frames (ORFs) are indicated in blue color with arrows indicating the ORF direction. The functional annotation of these ORFs in phages SPA01 and SPA05 is shown in Table S1. The data shown in (**C**,**D**,**G**,**H**) represent the mean ± standard deviation of at least triplicates.

The lytic phages named SPA01 and SPA05 were isolated from a canal in Bangkok, Thailand, using *P. aeruginosa* strain PAO1 as a parental bacterial host. Both phages were capable of forming 2–4 mm clear plaques on the PAO1 lawns (Fig. 1A,E), which the relatively large clear plaque appearance indicated the potent lytic activity of the phages against the bacteria. The morphology of phages SPA01 (Fig. 1B) and SPA05 (Fig. 1F), as examined by TEM and classified based on the criteria proposed by Ackermann^26^, revealed that both phages belong to the family *Myoviridae* due to the presence of an icosahedral head: 74 ± 9 nm and 75 ± 4 nm in diameter of SPA01 and SPA05 (n = 4), and a contractile tail: 163 ± 10 nm and 164 ± 5 nm in length of SPA01 and SPA05 (n = 4).

To investigate whether these phages are appropriate for medical and biotechnological purposes, their biological properties, including host range, adsorption rate, one-step growth curves, and phage tolerance were evaluated. The host range of phages against various *Pseudomonas* spp., including lab and clinical isolates, was first determined and the result showed that most of the clinical isolates were susceptible to phages SPA01 (10/13) and SPA05 (7/13) (Table 1). However, both phages were unable to infect the *P. stutzeri, P. mendocina, P. fluorescens,* and *P. putida* strains. This suggests that both phages are likely specific to a relatively wide spectrum among *P. aeruginosa* species, highlighting their potential use in medical treatment. To measure the infectivity of phages, the efficiency of plating (EOP) was performed. Of all strains tested, while none of them showed a higher EOP value than the indicator host strain, *P. aeruginosa* PAO1, the majority of EOP values obtained from phage SPA01 were higher than those obtained from phage SPA05 (Table 1), indicating that SPA01 exhibited higher infectivity towards tested strains.

**Table 1:** Bactericidal spectrum and efficiency of plating (EOP) of the phages.

Host	Source	SPA01*	SPA05*	PhiKZ*	PhiPA3*
*Pseudomonas aeruginosa* PAO1	Lab strain	1	1	1	1
*Pseudomonas aeruginosa PSU-PA01*	Clinical strain, Rectal swab	0.300	0.102	0.571	0.938
*Pseudomonas aeruginosa PSU-PA02*	Clinical strain, Rectal swab	–	–	–	–
*Pseudomonas aeruginosa PSU-PA03*	Clinical strain, Rectal swab	0.355	0.327	0.259	0.750
*Pseudomonas aeruginosa PSU-PA04*	Clinical strain, Throat swab	0.150	0.123	0.100	0.219
*Pseudomonas aeruginosa PSU-PA05*	Clinical strain, Rectal swab	0.682	0.545	0.714	0.875
*Pseudomonas aeruginosa PSU-PA06*	Clinical strain, Rectal swab	–	–	0.202	–
*Pseudomonas aeruginosa PSU-PA07*	Clinical strain, Throat swab	0.545	–	0.857	–
*Pseudomonas aeruginosa PSU-PA08*	Clinical strain, Throat swab	0.277	0.555	0.232	–
*Pseudomonas aeruginosa PSU-PA09*	Clinical strain, Rectal swab	0.409	0.227	0.639	0.750
*Pseudomonas aeruginosa PSU-PA10*	Clinical strain, Throat swab	0.241	0.318	0.625	0.813
*Pseudomonas aeruginosa PSU-PA11*	Clinical strain, Throat swab	0.591	–	0.563	0.938
*Pseudomonas aeruginosa PSU-PA13*	Clinical strain, Throat swab	–	–	–	–
*Pseudomonas aeruginosa PSU-PA14*	Clinical strain, Throat swab	0.727	–	0.448	1.063
*Pseudomonas aeruginosa* ATCC9027	American Type Culture Collection	–	–	–	–
*Pseudomonas aeruginosa* ATCC15442	American Type Culture Collection	0.104	0.418	0.116	0.168
*Pseudomonas aeruginosa* ATCC27853	American Type Culture Collection	0.186	0.154	0.142	0.106
*Pseudomonas stutzeri* DMST28410	Department of Medical Sciences Thailand	–	–	–	–
*Pseudomonas stutzeri* DMST12562	Department of Medical Sciences Thailand	–	–	–	–
*Pseudomonas mendocina* ATCC25411	American Type Culture Collection	–	–	–	–
*Pseudomonas fluorescens* ATCC13525	American Type Culture Collection	–	–	–	–
*Pseudomonas putida* ATCC12633	American Type Culture Collection	–	–	–	–
*Pseudomonas putida* ATCC17522	American Type Culture Collection	–	–	–	–

*Efficiency of plating (EOP) values were classified as highly productive (≥ 0.5), medium productive (0.1 ≤ EOP < 0.5), low productive (0.001 < EOP < 0.1) or inefficient (≤ 0.001).

In aspects of adsorption, the majority of phages SPA01 (90%) and SPA05 (92%) adsorbed to the bacterial host cells within 10–15 min (Fig. 1C,G), followed by a 30-min latent interval for replication inside the cells (Fig. 1D,H). Both phages then burst the bacterial host, which resulted in approximately 218–240 virions per cell (Fig. 1D,H), highlighting the potency of these phages in therapeutic aspects since phages with high burst sizes are favorable^27–29^. The phage tolerance test showed that both phages were relatively stable over a wide range of temperatures and pHs (Fig. S1A and S1B). The phages withstood the high temperature of 70 °C and did not lose much of their infectivity when exposed to acidic and basic conditions (pH 4–10), indicating the high viability of phages SPA01 and SPA05 as resilient biocontrol agents (Fig. S1A and S1B). Altogether, these promising characteristics of phages SPA01 and SPA05 render them potential candidates for both therapeutics and biocontrol of *P. aeruginosa*.

### Genomic characterization of phages SPA01 and SPA05

Apart from desirable phenotypic characteristics of phages that result in high infectivity against the targeted bacteria, genetic information is also crucial in selecting an appropriate, non-harmful phage for therapeutic use. To determine whether the undesirable genes associated with antibiotic resistance, bacterial virulence, and the lysogenic life cycle were present in the phage genomes, we conducted whole-genome sequencing, employed SPAdes^30^ for de novo assembly, and annotated all open reading frames (ORFs) throughout the phage genomes. Genome analysis revealed that phages SPA01 and SPA05 contained 93,536 bp and 93,656 bp of double-stranded DNA genome, respectively (Fig. 1I,J); therefore, both phages were considered relatively small phages due to the genome size of less than 200 kb^31^. The %GC contents of the SPA01 and SPA05 genomes were 49.34% and 49.49%, respectively. The schematic genome maps of phages SPA01 and SPA05 created by the CG view server^32^ showed the outer ring representing coding sequence locations (CDS) in blue and tRNAs in this ring in yellow. Genome annotation of phages using PHASTER^33^, GeneMark^34^, and RAST^35^ demonstrated that phages SPA01 and SPA05 encoded 171 and 175 ORFs, respectively. Among them, only 42 ORFs and 52 ORFs of phages SPA01 and SPA05 (TABLE S1) were identified as putative functional proteins. Fifteen tRNA-encoding genes were identified in both phages SPA01 and SPA05 using tRNAScanSE^36^. The tRNA functions in the phage genome are still not clear since the translation of viral proteins mostly depends on host tRNA^37,38^. According to Megablast results, SPA01 has a 97.3% sequence similarity to *Pseudomonas* phage PAK P1, while SPA05 has a 97.8% sequence similarity to *Pseudomonas* phage vB_PaeM_MAG1. Comparative intergenomic similarities by VIRIDIC analysis of phages SPA01 and SPA05 with other closely related phages revealed that both phage genomes showed high similarity to other *Pakpunavirus*, including PAK_P1^39^, K8^40^, PaP1^41^, JG004^42^, vB_PaeM_MAG1^43^, PAK_P2^39^, and vB_PaeM_C2 − 10_Ab1^44^ (Fig. S3). Moreover, the intergenomic similarities was below the threshold (95%) of identification of the similar species^50^. Therefore, these phages were classified as a novel phage in the genus “*Pakpunavirus*” (Fig. S3B). The ORFs were mainly annotated as structural proteins, nucleotide metabolism and DNA replication-related proteins, and bacterial lysis proteins, as indicated in Table S1. Importantly, undesirable genes involved in bacterial virulence, toxins (exotoxin A, pyocyanin, hydrocyanic acid, enterotoxin etc.), or lysogeny were not identified within the phage genomes, assuring the lytic nature and implying safety for use of these two phages.

### Potential of phages SPA01 and SPA05 in *P. aeruginosa* suppression and formation of phage-resistant bacteria

**Figure 2:**
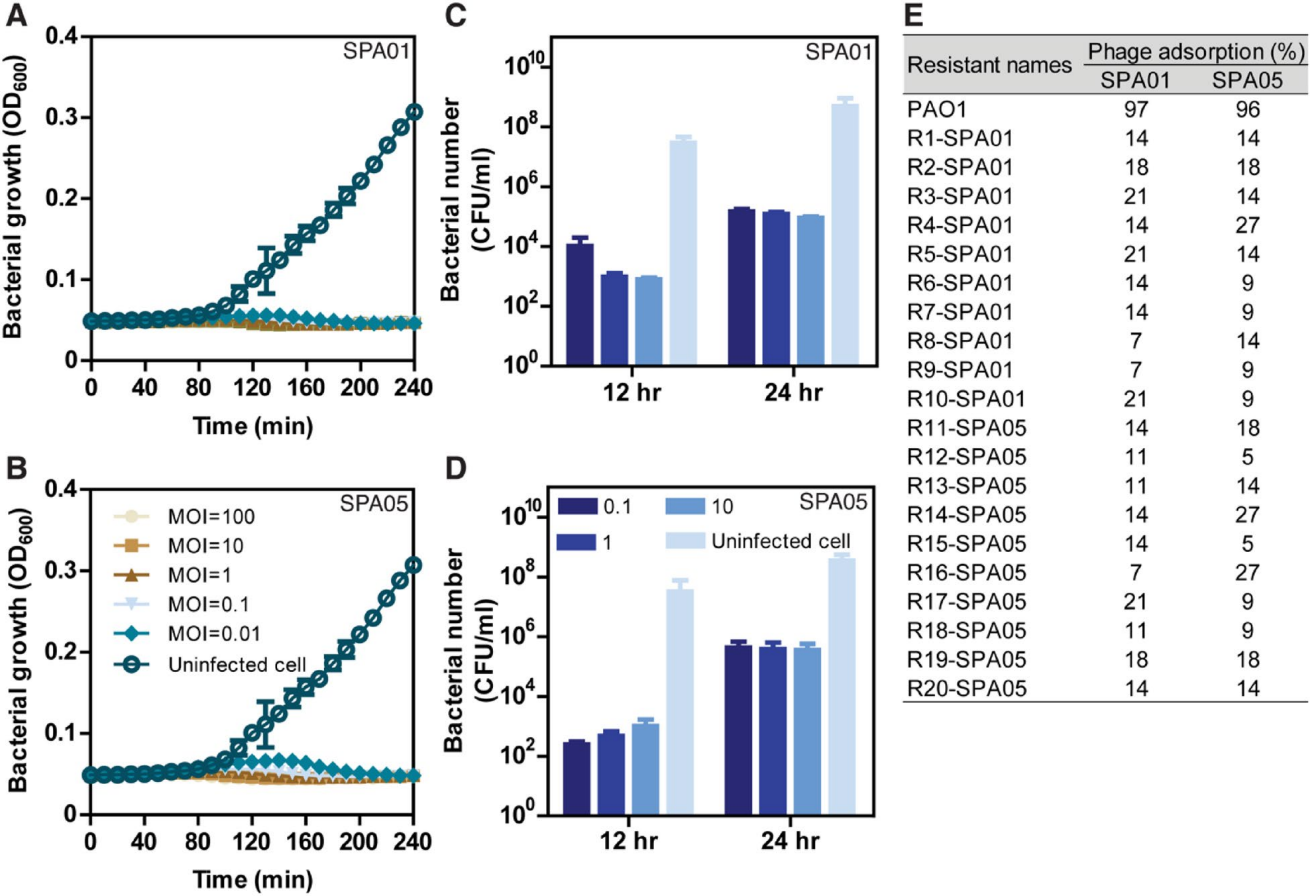
Potential of phages SPA01 and SPA05 in *P. aeruginosa* suppression and formation of phage-resistant bacteria. Killing profiles of phages SPA01 (**A**) and SPA05 (**B**) against *P. aeruginosa* strain PAO1 in vitro at MOIs of 0.01, 0.1, 1, 10, and 100. Survival *P. aeruginosa* PAO1 (CFU/ml) in the presence of SPA01 (**C**) and SPA05 (**D**) for 12 and 24 h, showing the increasing emergence of phage resistance through time. Quantitation of adsorption of phages SPA01 and SPA05 among phage resistant strains, suggesting cross resistance to phages through mutations of bacterial receptors (**E**). Unabsorbed phage titers were measured after incubation with each phage-resistant strain: SPA01-resistant isolates (R1 to R10-SPA01) and SPA05-resistant isolates (R11 to R20-SPA05). The data shown in (**A–D**) represent the mean ± standard deviation of at least triplicates.

Since both phages SPA01 and SPA05 exhibited desirable characteristics of a good candidate for bacterial growth control, an *in vitro* lysis assay was performed to evaluate the lytic activity of phages SPA01 and SPA05 against *P. aeruginosa* at different multiplicity of infections (MOIs). The results showed that both phages displayed efficient killing activity against the bacteria, even at the very low MOI of 0.01 (Fig. 2A,B). Although no bacterial regrowth was observed throughout a window of 240 min in both phages, viable bacteria could be detected at a longer timepoint (Fig. 2A–D), suggesting the emergence of phage-resistant bacteria. SPA01 and SPA05 resistant *P. aeruginosa* isolates, in particular, were observed at 12 h and found to be approximately 2-log increased at 24 h in all tested MOIs (Fig. 2C,D). The revival frequency of bacteria toward phages SPA01 and SPA05 was quite comparable, which equals 0.0015–0.0017. This finding is unsurprising since it has been well documented that the use of phage, especially in vitro studies, normally leads to an early emergence of resistance^45–47^. Notably, despite resistance emergence during phage treatment, some studies have shed light on the possible evolutionary tradeoff of phage-resistant strains in *P. aeruginosa* that can lead to attenuation of bacterial virulence^46,48^ or hypersensitivity to antibiotics^49,50^ that would be beneficial for following treatments.

To further explore what possible resistance mechanisms the hosts exhibit against phages SPA01 and SPA05, we first isolated 10 *P. aeruginosa* strains that were resistant to each of the phages and examined the host adsorption rate, which is one of the most common anti-phage mechanisms^51^. Interestingly, the percentage of adsorption of each phage to all corresponding phage-resistant strains substantially dropped (Fig. 2E), suggesting that the mechanism of phage resistance in these strains possibly occurs via receptor modification. However, since phage resistance mechanisms can occur through the host immune system or genetic modification^9,52,53^, further investigation will be required to explore other possible resistance mechanisms in these isolates. We then investigated whether phage resistance to one phage affects susceptibility to another phage by measuring phage adsorption when cross-infected with the resistant isolates. The results demonstrated that all SPA01-resistant isolates were also resistant to phage SPA05 and vice versa, as indicated by the decreased phage adsorption percentage on the resistant strains (Fig. 2E). This finding suggested that these phages are closely related and infect the bacterial host through a similar adsorption mechanism. It has been shown that type IV pili (T4P) and lipopolysaccharide (LPS) O antigen are the common binding sites for *P. aeruginosa* phages^39–42,54,55^. Our VIRIDIC data revealed that phages SPA01 and SPA05 are classified as *Pakpunavirus* (Fig. S3). Therefore, they are assumed to recognize LPS during the adsorption step, similar to other previous studied *Pakpunavirus* (Table S2). Thus, the impairment of phage adsorption in these resistant strains might affect the efficiency of the phage cocktail that is composed of SPA01 and SPA05. However, further investigation into the receptor, which serves a role in phage recognition, is needed to gain insights into the adsorption step of phages SPA01 and SPA05.

It is well known that closely related phages are not compatible candidates for being the major components in a phage cocktail due to the possibility of cross resistance, which could jeopardize the cocktail's overall effectiveness. In order to diminish the emergence of phage-resistant bacteria, one of the strategies for cocktail design is to mix phages that are genetically divergent as they are assumed to display different replication mechanisms, such as targeting different host range and hijacking different bacterial molecular targets, particularly in this study as an example, recognizing different host cell receptors. This idea was previously supported by a study demonstrating that a combination of 5 genetically diverse phages against *Mycobacterium tuberculosis* greatly reduced persistence and phage-resistant bacteria^56^. However, insights into the replication machinery of these 5 genetically diverse *Mycobacterium* phages has still been unexplored.

### Phages SPA01 and SPA05 are genetically and mechanistically diverse from jumbophages PhiKZ and PhiPA3

**Figure 3:**
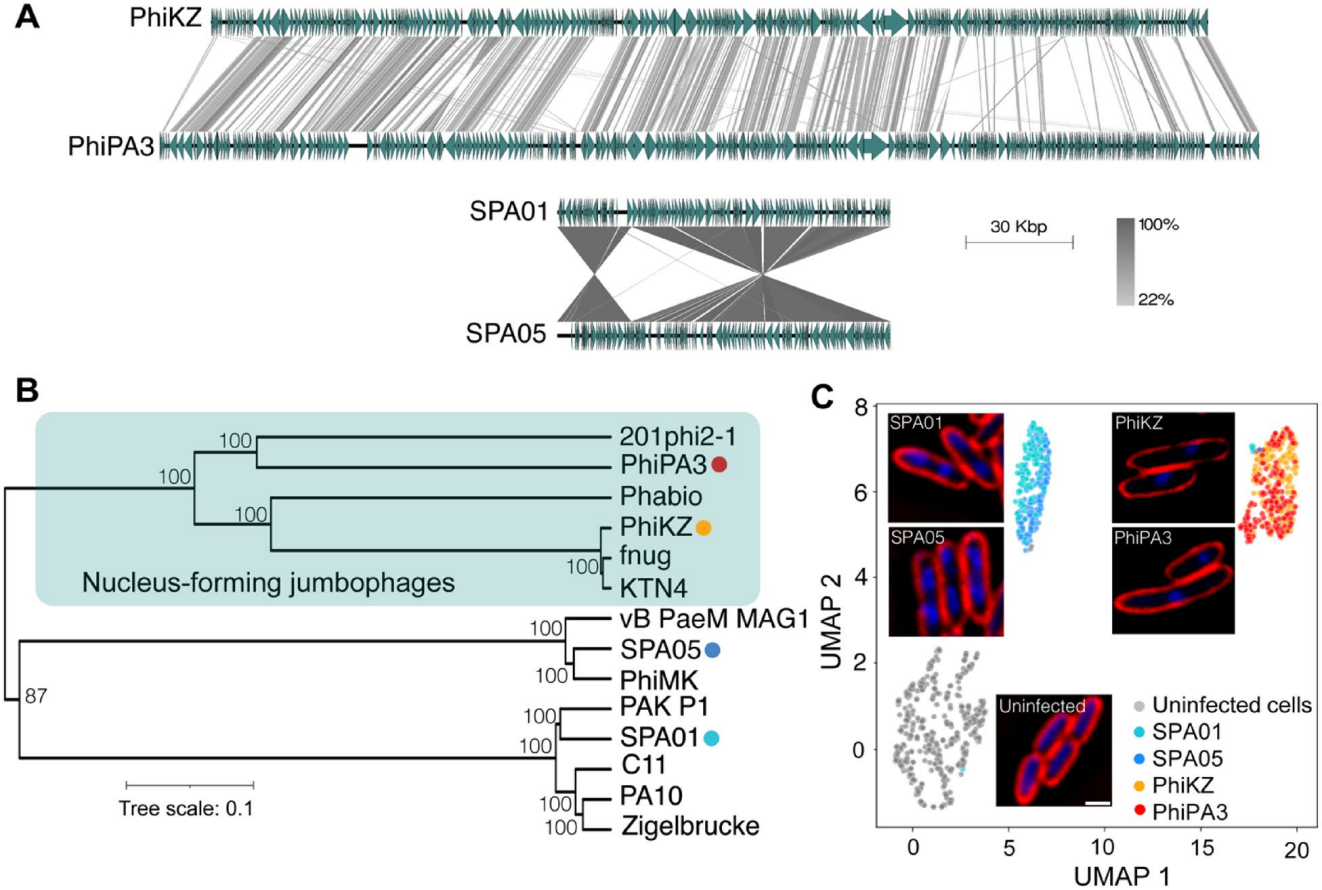
Phages SPA01 and SPA05 are genetically and mechanistically diverse from the nucleus-forming jumbophages. Comparative genome analysis of small phage genomes (SPA01 and SPA05) with giant phage genomes (PhiKZ and PhiPA3) (**A**). The green arrows represent the coding sequence directions and locations, and gray shaded lines reflect the degree of homology between them. Phylogenetic tree based on whole-genome sequence comparisons of selected phages, generated with Geneious version 2022.2.2 using the neighbour-joining method and visualized with iTOL (**B**). The single-cell level assay reveals the bacterial morphological changes triggered during infections of small (SPA01 and SPA05) and giant (PhiKZ and PhiPA3) phages at MOI 5. *P. aeruginosa* cells at OD_600_ ~ 0.4 were infected with phages at MOI 5 and fixed at 20 min post infection (mpi), followed by staining cell membrane with FM4–64 (red) and nucleoid with DAPI (blue). Scale bar equals to 1 micron (**C**). The cytological profile was performed by Uniform Manifold Approximation and Projection (UMAP), showing cell clusters of uninfected cells (gray) and cells infected with small phages (SPA01; light blue, and SPA05; blue) and jumbophages (PhiKZ; orange, and PhiPA3; red).

With the previous evidence that genetically diverse phages might serve roles in prolonging the emergence of phage resistance in the phage cocktail^56^, we reasoned that nucleus-forming *Pseudomonas* jumbophages that are believed to display a complicated infection machinery and could recognize a different phage receptor on the host cells would be beneficial as a candidate for the cocktail with phages SPA01 and SPA05. We first confirmed whether they are genetically different and investigated how they are phylogenetically clustered. The whole genome alignment of the phages demonstrated that the phage PhiKZ genome has a high degree of similarity in genome organization to that of phage PhiPA3, while the genomes of the jumbophages are organized differently from the genomes of the phages SPA01 and SPA05 (Fig. 3A). Phylogenetic analysis of the whole genome sequence further revealed that phages PhiKZ and PhiPA3 were closely related and grouped together in the clade of nucleus-forming jumbophages and speciated from other small phages, including phages SPA01 and SPA05 (Fig. 3B). Due to the large difference in genome size and organization among the phages, as expected, this result confirmed that our phages SPA01 and SPA05 are genetically diverse from the jumbophages PhiKZ and PhiPA3.

The viral replicating or hijacking machinery, as we called it “Mechanism of pre-killing (MOK)”, can be studied through the bacterial morphology that is triggered during phage infection. Through the morphological change, we have investigated how nucleus-forming jumbophages replicate inside bacteria^57–60^ and predicted the bacterial fundamental metabolism that was interfered by phages^61^. Since we speculated that the small phages SPA01 and SPA05 might exhibit a distinct replication process during infection differing from the jumbophages PhiKZ and PhiPA3, as corresponded to their genetic divergence, we performed a single cell infection assay to observe if the morphological changes as triggered during the infection by these phages are different. The result revealed that all phages induced morphological changes in bacteria when compared to uninfected cells (Fig. 3C). In particular, SPA01- and SPA05- infected cells contained two compact nucleoids, while PhiKZ- and PhiPA3- infected cells were slightly longer and contained only one condensed nucleoid close to midcell (Fig. 3C). The morphology of PhiKZ- and PhiPA3- infected cells was well in agreement with our previous studies that revealed the presence of the phage nucleus that is centrally located near midcell during jumbophage infection ^62^. We next quantitated the cytological profiles of each group at the single-cell level using morphological parameters reported previously ^63,64^ to test if the morphological changes caused by each phage are separated or clustered together. The result showed that SPA01- and SPA05-infected cytological profiles are clustered together and separated from PhiKZ- and PhiPA3-infected cells (Fig. 3C), suggesting that the two groups of phages trigger different morphological changes of cells during infection, possibly through different MOKs. Further studies will be needed to gain better understanding regarding MOKs of phage SPA01 and SPA05, in which it will provide insights into correlation between the MOK and the apparent morphological change. In summary, this evidence agrees with the evolutionary divergence of the phages, thus reassuring that these phages (SPA01 and SPA05) and the nucleus-forming jumbophages are divergent.

### The combination of the nucleus-forming jumbophages with either phages SPA01 or SPA05 efficiently suppresses the growth of bacteria and reduces the frequency of bacterial revival

**Figure 4:**
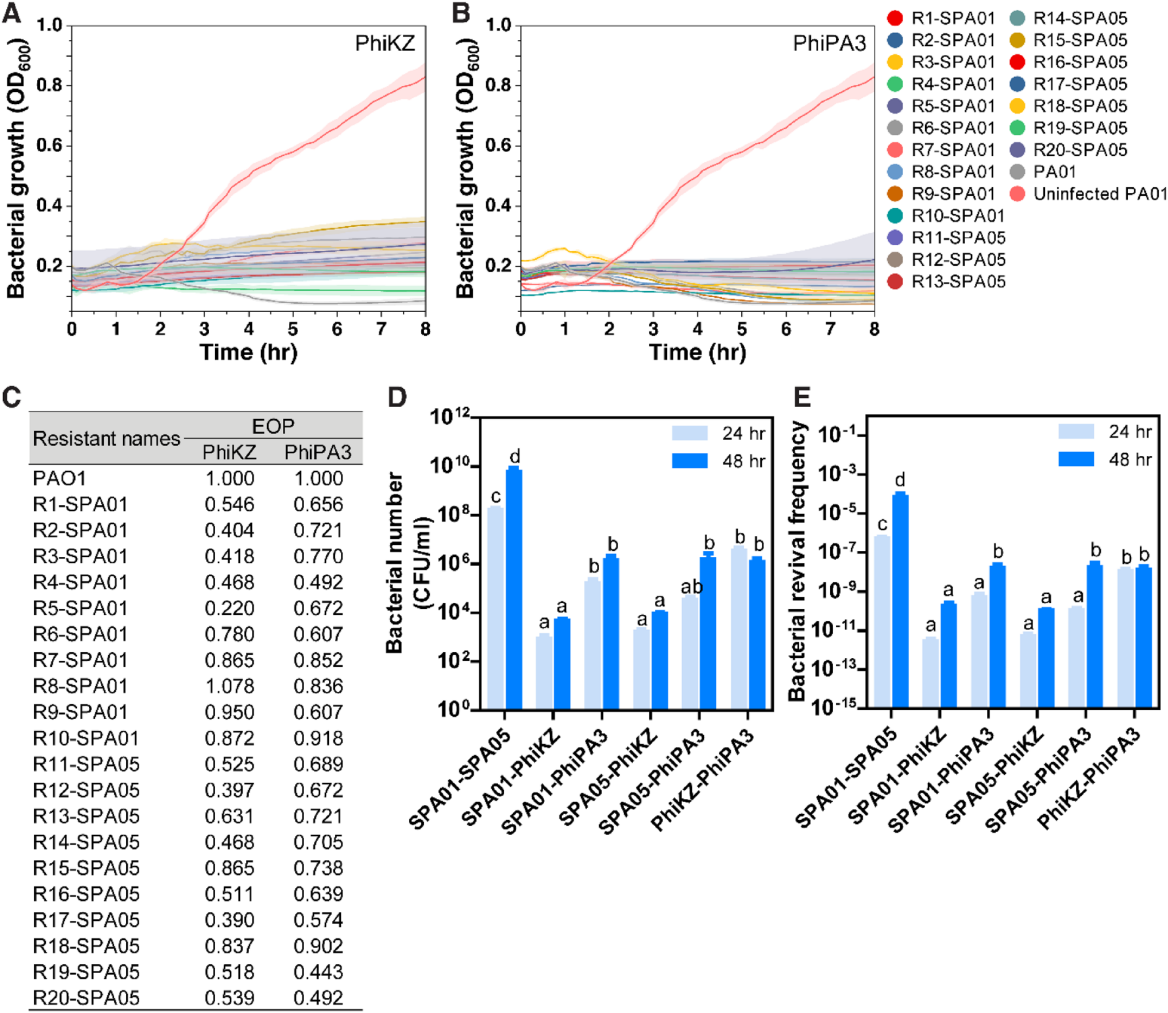
Giant phages PhiKZ and PhiPA3 efficiently suppress the growth of SPA01 and SPA05-resistant bacteria and improve the efficacy of the phage cocktail. Killing profiles of phages PhiKZ (**A**) and PhiPA3 (**B**) in all SPA01 and SPA05-resistant strains (R1 to R10-SPA01 and R11 to R20-SPA05) at a MOI of 1, indicating the susceptibility of small phage-resistant strains to giant phages. EOP of phages PhiKZ and PhiPA3 in all SPA01 and SPA05-resistant strains compared to *P. aeruginosa* PAO1, suggesting medium to high production of giant phages in the resistant isolates (**C**). Survival *P. aeruginosa* PAO1 (CFU/ml) at 24 and 48 h in six different formulas of phage cocktails: (1) SPA01-SPA05, (2) SPA01-PhiKZ, (3) SPA01-PhiPA3, (4) SPA05-PhiKZ, (5) SPA05-PhiPA3, and (6) PhiKZ-PhiPA3 (**D**). The revival frequency of *P. aeruginosa* PAO1 at 24 and 48 h in six different formulas of phage cocktails (**E)**. The data shown in (**A-E**) represent the mean ± standard deviation of at least triplicates. Statistical significance in (**D-E**) was calculated by two-way ANOVA. Different letters above bars show values that are significantly different (*p* < 0.05).

During replication, PhiKZ-like viruses exhibit an orchestrated infection machinery by encoding a tubulin homolog named PhuZ to organize a nucleus-like compartment at midcell and deliver procapsids to dock on the phage nucleus surface for encapsidation^57–60,65,66^. The phage nucleus serves a role in partitioning proteins according to function and in protecting the phage nucleic acids against broad bacterial DNA-targeting immune systems such as clustered regularly interspaced short palindromic repeats (CRISPR) systems and restriction-modification (R-M) systems^65–67^. Due to their advantages over the small phages, as mentioned here, together with the idea of combining them with the phages SPA01 and SPA05 to formulate an efficient cocktail, we first tested whether the nucleus-forming jumbophages could lyse the phage SPA01 or SPA05-resistant isolates. Strikingly, both giant phages were able to kill all isolates that were resistant to each small phages SPA01 and SPA05, and efficiently suppressed the bacterial growth for at least 8 h (Fig. 4A,B). Results of the EOP also showed the high efficiency of the giant phages to lyse the resistant isolates in addition to their host bacteria (Fig. 4C). Seventeen phage-resistant isolates produced high levels of phage PhiPA3 production, having EOPs greater than 0.5, while thirteen strains could be classified as having high phage PhiKZ production (Fig. 4C). These results suggested that the giant phages (PhiKZ and PhiPA3) recognize different receptors on the target cells from those small phages (SPA01 and SPA05), making them a potential candidate for the phage cocktail formula.

Due to the therapeutic potential of giant phages PhiKZ and PhiPA3 as they kill various clinical *P. aeruginosa* strains (Table 1, Fig. S2) and are capable of infecting the phage SPA01 and SPA05-resistant isolates (Fig. 4A–C), various combinations between the giant phages and the small phages (SPA01 and SPA05) were then formulated to make phage cocktails. The cocktail mixtures included 6 formulas as follows: (1) SPA01-SPA05, (2) SPA01-PhiKZ, (3) SPA01-PhiPA3, (4) SPA05-PhiKZ, (5) SPA05-PhiPA3, and (6) PhiKZ-PhiPA3. The cultures of *P. aeruginosa* PAO1 were grown in the presence of the phage cocktails at MOI 1 and the viable bacterial number was counted at 24 h and 48 h. The result showed that the remaining bacterial number that survived through the phage treatment was detectable in all formulas, suggesting the presence of phage resistance isolates in all conditions. However, the number of viable bacteria when treated with the small phage cocktail (SPA01-SPA05) was around 4 to 6 -log higher than those when treated with the combinations that contained a giant phage. The SPA01-SPA05 resistant bacteria outgrew faster than the others, with an around 2-log increase from 24 to 48 h (Fig. 4D). This result suggested that the closely related phages are not an appropriate combination in the cocktail since resistance to one might result in cross-resistance to another. Interesting, adding either one of the giant phages into the cocktail could efficiently suppress the regrowth of bacteria, as the viable bacterial numbers detected at both time points were significantly reduced when compared to the small phage cocktail. Furthermore, adding the giant phages, particularly PhiKZ, to the combination completely suppressed the bacterial regrowth, resulting in a comparable bacterial number at 48 h to 24 h, except for the cocktail SPA05-PhiPA3 (Fig. 4D).

The revival frequency of *P. aeruginosa* PAO1 treated with each phage cocktail was then investigated to evaluate the durability of phage therapy. The results showed that the frequency of revival developed in the small phage cocktail significantly increased from 24 to 48 h. The increase in revival frequency was also observed in the cocktails containing PhiPA3, whereas the increased revival was not observed in the cocktails that included PhiKZ (Fig. 4E), indicating that the giant phage PhiKZ is an appropriate ingredient for the cocktail with the small phages. Additionally, the cocktail of giant phages (PhiKZ-PhiPA3) alone also gave a promising outcome as it suppressed the bacterial regrowth from 24 to 48 h with unchanged revival frequency at 48 h when compared to the small phage cocktail (Fig. 4D,E). However, it is worth noting that, due to the survival of bacteria during the treatment of the PhiKZ-PhiPA3 combination that was significantly higher than that of the combinations of small and giant phages (Fig. 4D), this might be a result of co-infection of these closely related jumbophages in bacteria that might impair their infectivity as they interfered with each other through virogenesis incompatibility^68^.

It has long been known that phage cocktails consisting of different or distantly related phages are more effective in inhibiting the growth of targeted bacteria^69–72^. This is, in part, because different phages usually possess different mechanisms during replication^11^; therefore, multiple resistances are required for the bacteria to fend off the infections caused by various phages in the cocktail. Thus, as observed here, combining the diverse phages that recognize different receptors on the target cells during phage entry in the cocktail can enhance the efficacy of the cocktail and decelerate the emergence of phage resistance.

### Implications for phage cocktail design

Conventional criteria for choosing phage cocktails are mainly based on empirical testing of different combinations of phages. Despite the fact that various methods for accelerating the process, such as high-throughput phage combination studies^73^, laborious work are still required for studying individual phage biology in order to properly select the phages for cocktail. Recently, the rise of genomic research and bioinformatic tools facilitates biologists in dissecting phage biology based on DNA sequence, which serve as a navigator toward choosing the right mix of diverse phages into cocktails^74^. However, the selection of phages for the cocktail is still largely based on genomic data. Here, we propose a complementary perspective for phage cocktail formulation via MOK-guided phage selection to choose genetically diverse phages for rapid cocktail design without the need for phage genomic data. Based on the morphological changes of bacterial cells triggered during phage infection together with the quantitation of the cytological profiles at a single-cell level, this information will provide additional criteria to the current phage selection checklist.

Although these MOK-based approaches through morphological changes would be beneficial in the selection of diverse phages for the rapid design of effective phage cocktails, there are some limitations in this study worth noting. First, not all phages could induce phenotypic changes of bacteria upon infection. Since our reports of MOKs in nucleus-forming jumbophages and a vibriophage, only a handful of studies have reported unique MOKs observed in other phages and their hosts, resulting in limited information regarding possible MOKs. Thus, to test whether this finding could be broadly applied to phage cocktail design, more systematic studies of MOKs in various phage-host systems are needed. Second, while we were able to distinguish different types of phage-induced morphological changes at the single cell level, we were unable to pinpoint which mechanisms the phages used inside the bacterial cell to cause detectable cytological changes at the molecular level. Future studies on whole genome sequence of phage resistant bacteria that abolish cell morphology alteration will be critical in providing insight into the molecular targets of phage infection and thus bridging the knowledge gap between cytological response and molecular mechanism upon phage infection. Lastly, cytological changes of phage-infected cells could vary through each time point after infection, and synchronizing infection time is challenging, especially in a single cell study; thus, time course experiments are needed to truly understand the mechanism by which phage deploys during infection. Information regarding the temporal MOK of phages in the cocktails will also serve as a key to dissecting phage-phage interaction for the better development of therapeutic phage cocktails in the future.

## Conclusion

We successfully isolated 2 virulent *Pseudomonas* myophages, SPA01 and SPA05, that exhibited potential therapeutic characteristics against various clinical strains of *P. aeruginosa*. Even though the phages efficiently suppressed the bacterial regrowth within 4 h, the phage SPA01 and SPA05-resistant bacteria subsequently emerged, possibly due to modification of a receptor where the phages bind during adsorption. The nucleus-forming jumbophages (PhiKZ and PhiPA3) that are distantly related to the phages SPA01 and SPA05 were thus selected as a potential candidate for formulating the phage cocktails. Through various cocktail formulas, we demonstrated that combining the jumbophages PhiKZ with either of the small phages improved the overall efficiency of the cocktail by better suppressing bacterial regrowth and delaying the emergence of phage resistance.

## Materials and methods

### Bacterial strains and growth conditions

Twenty-three *Pseudomonas* strains were employed in this study including 17 *P. aeruginosa* strains, 2 *P. stutzeri* strains, 2 *P. putida* strains, *P. mendocina* ATCC25411, *P. fluorescens* ATCC13525. A complete list of *Pseudomonas* strains can be found in Table 1. *P. aeruginosa* strains *PSU-PA01–PSU-PA14*, isolated from patients in internal medicine, Songklanagarind hospital, Thailand, were a generous gift from the RP lab. All bacteria were grown on LB broth (10 g/l tryptone, 10 g/l NaCl, and 5 g/l yeast extract) at 37°C followed by shaking at 250 rpm. When plated, cells were grown on LB containing 1.5% agar and incubated inverted at 37°C.

This work has been reviewed and approved by Chulalongkorn University-Institutional Biosafety Committee (CU-IBC) in accordance with the levels of risk in pathogens and animal toxins, listed in the Risk Group of Pathogen and Animal Toxin (2017) published by Department of Medical Sciences (Ministry of Public Health), the Pathogen and Animal Toxin Act (2015) and Biosafety Guidelines for Modern Biotechnology BIOTEC (2016) with approval number: SC CU-IBC-028/2020 Ex1.

### Phage isolation, amplification, host range, and transmission electron microscopy

Water samples were collected from Khlong Samrong Bangkok, Thailand. Briefly, filtered water sample through a 0.45 µm membrane was mixed with 2X LB medium and then added log phase culture of *P. aeruginosa* PAO1 followed by incubation at 37 °C with shanking at 250 rpm for 24 h. After centrifugation, the supernatant was mixed with LB containing 0.5% agar powder and subsequently combined with the host. The mixture was overlaid onto LB plate and incubated at 37 $^\circ$C for 24 h to observe plaque formation. The isolated plaque was collected and re-plated five times to ensure single phage purification. The purified phages were designated as phages SPA01 and SPA05. Phage stocks were maintained in SM buffer with gelatin (100 mM NaCl, 8 mM MgSO_4_·7H_2_O*,* 50 mM Tris–Cl, pH 7.5 caution 0.01% (w/v) gelatin) and subsequently stored at 4 $^\circ$C.

For phage propagation, 20 ml of the host culture (1 × 10^6^ CFU/ml) was infected at a multiplicity of infection (MOI) of 1 and incubated for 24 h at 37 $^\circ$C. The host cell was removed by centrifugation. The supernatant was filtrated (0.45 µm filter) and subsequently stored at 4 $^\circ$C for further experiments. To determine phage titration, a tenfold serial of phage stocks was mixed with the host culture and poured into an LB soft agar plate. The plate was incubated overnight at 37 °C. The number of phages was enumerated by plaque observation and expressed as plaque forming unit (PFU) per ml.

Host range was identified by 23 *Pseudomonas* strains. The bacterial lawn was prepared with the bacteria and then 5 µl of phages were dropped onto the lawn of each host. The plate was incubated at 37 $^\circ$C for 24 h to observe the lytic zone. The experiment was undertaken in triplicates. The efficiency of plating (EOP) values is calculated as the ratio of the PFU value of phage with susceptible bacterial strain and the phage with indicator bacterial strain.

Transmission electron microscopy (TEM) was undertaken to visualize phage morphology by negative staining method ^75^. Briefly, fresh plaques were collected and suspended in SM buffer. The supernatant was placed on copper grids and negatively stained with 2% (w/v) uranyl acetate (pH 4.5). Phage morphology was observed with a Hitachi HT7700 transmission electron microscope at 80 kV.

### Phage adsorption and one-step growth curve

The phage adsorption curve was determined by growing host cells to log phase (~ 1 × 10^8^ CFU/ml), then infected with phages at an MOI of 0.01, and incubated at 37 °C. Aliquots of 1 ml were taken at an interval of every 5 min for 25 min and filtrated through 0.45 µm. The titer of unabsorbed phages was calculated from filtrates.

One-step phage growth curve was performed. Five milliliters of log phase host culture were centrifuged and resuspended in 1 ml of LB. Phages were transferred at an MOI of 0.01 and incubated at room temperature for 15 min for phage adsorption. The pellet containing infected cells was harvested and resuspended in 20 ml of LB broth and then incubated at 37 °C. Samples were taken at 10 min intervals for 70 min and subsequently filtrated through 0.45 µm. The titer of phages was immediately determined using the double-layer technique. This experiment was undertaken in triplicate. The latent period, burst time, and burst size of the phage were estimated as described elsewhere^76^.

### Effects of temperature, and pH on phage viability

Stability of phage at different conditions in SM buffer was determined. The various temperature (25 $^\circ$C, 30 $^\circ$C, 37 $^\circ$C, 40 $^\circ$C, 50 $^\circ$C, 60 $^\circ$C and 70 $^\circ$C) and pHs (2, 4, 6, 8, 10, and 12) were prepared by diluting phage stock to a final concentration of 10^6^ PFU/ml in 1 ml of SM buffer for 2 h. Subsequently, phage titers were measured as described above. The experiments were carried out in triplicate.

### Phage lytic ability and phage-resistant bacterial outgrowth

Killing curves optimal MOI was determined using phages on PAO1. The bacterial host was infected with phage suspension to give an MOI of 0.01, 0.1, 1, 10, and 100. The log-phase bacterial cells were loaded into 96-well microplate and mixed with different MOIs of phage in 200 μl LB medium. The optical density of the plate was measured automatically every 10 min intervals up to 240 min at a wavelength of 600 nm. To verify the antibacterial efficacy of phage, bacterial number were counted after infection at MOI of 0.1, 1, and 10. Aliquots were removed at the indicated sampling time (12 and 24 h). Each independent trial was repeated three times.

To screen phage-resistant strains, *P. aeruginosa* PAO1 was grown at 37 $^\circ$C in LB broth. When the bacterial culture reached OD_600_ of 0.4, 10 µl of culture was mixed with 90 µl and 10^10^ pfu/ml of each phage. The phage-infected culture was spread to LB agar plate. Then, plates were grown at 37 $^\circ$C for 24 h. Ten randomly selected isolates were passaged five times on LB agar plate to confirm the stability of genetic changes. Resistance to SPA01 and SPA05 was confirmed in isolated clones by spot-test phage typing using phage suspension. Furthermore, the resistant strains were also tested the same way in terms of the susceptibility and the EOP to giant phages phiKZ and phiPA3. All tests were performed in triplicate.

### Single-cell infection assay and image data analysis

The PAO1 was cultured to reach OD_600_ ~ 0.4 and infected with phages at MOI of 5 followed by incubation for 20 min at 37 °C. The infected cells were fixed at a final concentration of 4% paraformaldehyde and incubated at room temperature for 15 min as described by Chaikeeratisak *et al*^57^. The fixed cells were harvested by centrifugation and then washed with 1 ml of 1 × PBS two times. The cells were resuspended in 1 × PBS and strained with fluorescent dyes (2 μg/ml FM 4–64 and 2 μg/ml DAPI). After centrifugation, 3 μl of cell suspension was loaded onto an agarose pad (1.2% agarose in 20% LB). The samples were visualized under Delta Vision Ultra High-Resolution Microscope.

Raw images from the fluorescent microscope were pre-processed on ImageJ software^77^. Then all individual cells were extracted morphological features by automatic cell analysis using Cellprofiler 4.0 software^78^. The morphological features set were selected based on the previous study^63^. Statistical calculation and Machine learning are carried out using scikit-learn library^79^ in python. Briefly, cell profile data were transformed and normalized with Cube Root Transformation and Z-scored normalization^80^, respectively. Finally, the dimension of the dataset was reduced and visualized with Uniform Manifold Approximation and Projection (UMAP)^81^. To compare the uninfected and infected cell profiles, the uninfected cell of the phage treatment group was excluded by selecting the infected cell cluster on the UMAP plot that didn’t overlap with the uninfected cell cluster.

### The bactericidal effect of the phage cocktail

The bactericidal ability of the 6 phage cocktails was assessed comprising (1) SPA01-SPA05, (2) SPA01-PhiKZ, (3) SPA01-PhiPA3, (4) SPA05-PhiKZ, (5) SPA05-PhiPA3, and (6) PhiKZ-PhiPA3. The log-phase of PAO1 was infected with phage cocktail at MOI of 1 followed by incubation at 37 °C. Samples were collected for measurement of number at 24 and 48 h. Each independent trial was repeated three times.

### Determination of bacterial revival frequency

The revival frequency of the bacteria was determined by a previous report with modification^82^. The PAO1 suspension was co-cultured with single phage or phage cocktail overnight at 37 °C with shaking at 150 rpm. The bacterial control without phages was incubated exactly as the infected cells. The number of bacteria was calculated based on the result of colony counting. The revival frequency was calculated using mean numbers of mutants in test samples divided by mean total numbers of control samples. Each independent experiment was repeated three times.

### Phage genome sequencing and analysis

The phage DNA were extracted using a previous report with modification^83^. Briefly, purified phage was treated with DNaseI (2 mg/ml) and RNaseA (100 mg/ml) overnight at 37  $^\circ$C. Subsequently, the lysis buffer (1 M Tris, pH 8.0, 0.5 M EDTA, pH 8.0, 10% SDS, and 10 mg/ml proteinase K) were added and incubated at 60 $^\circ$C for 1 h. The phage DNA was extracted twice with phenol: chloroform: isoamyl alcohol (25:24:1) and chloroform. After centrifugation, the supernatant containing DNA was collected and precipitated by mixing of a 0.3 volume of 3 M NaOA and 1 volume of isopropanol followed by incubation for 2 h at − 20  $^\circ$C. After the pellet was washed with 70% ethanol followed by centrifugation. The pellet was air-dried, dissolved with sterile distilled water, and then kept at −20 $^\circ$C.The DNA concentration was estimated based on absorbance 260 by the nanodrop. The absorbance 260/280 ratio and agarose gel electrophoresis were performed to evaluate DNA quality and integrity.

The whole-genome sequencing of phage DNA was performed using short-read sequencing through MGISEQ-2000 (Beijing Genomics Institute, Beijing, China). The quality of reads was checked with FASTQC^84^ and filtered using Trimmomatic 0.39^85^ with default parameters. Spades 3.11.1^30^ was used to assemble all reads into contigs. The open reading frames (ORFs) were identified using PHASTER^33^ and GeneMarkS^34^ following by annotation with Blastp of NCBI server. Antibiotic genes and virulence factors were identifed using ResFinder and VirulenceFinder^86^. The genome map of phages was drawn using the CG viewer server (http://cgview.ca/)^32^. The genomic comparison of phages with closely related *Pseudomonas* phages was illustrated in the form of a linear figure using Easyfig application (http://mjsull.github.io/Easyfig/fles.html)^87^. Furthermore, tRNAs were predicted tRNAscan-SE (http://lowelab.ucsc.edu/tRNAscan-SE/)^36^. The whole genome sequences were aligned in MAFFT online service^88^. The phylogenetic tree was generated in Geneious software (version 2022.2.2) using neighbour-joining method with 1000 bootstrap replications and visualized with Interactive Tree Of Life (iTOL)^89^.

### Statistics

Experiments were subject to two-way ANOVA with SPSS version 28.0.1 (SPSS, Inc., Chicago, IL, USA).

## Supplementary Information

Supplementary Information.

## Data Availability

Phage genome sequences of SPA01 and SPA05 were deposited in GenBank under the accession numbers OP875100.2 (www.ncbi.nlm.nih.gov/nuccore/OP875100) and OP875101.1 (www.ncbi.nlm.nih.gov/nuccore/2419105936), respectively.
