# Supplementary material for: Combination of genetically diverse Pseudomonas phages enhances the cocktail efficiency against bacteria
Source: Sci Rep. 2023 Jun 1;13:8921. doi: 10.1038/s41598-023-36034-2 (PMC10235106; doi:10.1038/s41598-023-36034-2)
Supplement: Supplementary file 1 — Supplementary Information. [file 41598_2023_36034_MOESM1_ESM.docx]

**TABLE S1** Putative functional proteins of phages SPA01 and SPA05

| **Phage SPA01** | | | | |
| --- | --- | --- | --- | --- |
| **ORF no.** | **Putative function** | **Sequence similarity** | **GenBank ID** | **E-value** |
| 16 | u-spanin | PHAGE_Pseudo_K5 | NC_030910 | 1.73E-125 |
| 17 | endolysin | PHAGE_Pseudo_vB_PaeM_C2_10_Ab02 | NC_042113 | 4.65E-135 |
| 18 | holin | PHAGE_Pseudo_K5 | NC_030910 | 3.99E-114 |
| 20 | u-spanin | PHAGE_Pseudo_Zigelbrucke | NC_041904 | 3.84E-97 |
| 25 | putative phosphoesterase | PHAGE_Pseudo_Zigelbrucke | NC_041904 | 3.98E-66 |
| 27 | D14 protein | PHAGE_Pseudo_phiMK | NC_031110 | 1.38E-07 |
| 30 | nicotinamide mononucleotide transporter | PHAGE_Pseudo_PA10 | NC_041903 | 4.80E-29 |
| 31 | HNH endonuclease | PHAGE_Pseudo_vB_PaeM_C2_10_Ab02 | NC_042113 | 1.43E-131 |
| 32 | NrdH family redoxin | PHAGE_Pseudo_PA10 | NC_041903 | 2.94E-78 |
| 34 | D11 protein | PHAGE_Pseudo_vB_PaeM_MAG1 | NC_031073 | 7.40E-41 |
| 35 | HNH endonuclease | PHAGE_Pseudo_Zigelbruck | NC_041904 | 6.40E-72 |
| 36 | HNH endonuclease | PHAGE_Staphy_phiSA012 | NC_023573 | 1.42E-10 |
| 37 | tail assembly protein | PHAGE_Pseudo_Zigelbrucke | NC_041904 | 3.97E-16 |
| 41 | TraG-like protein | PHAGE_Pseudo_PA10 | NC_041903 | 1.61E-110 |
| 43 | DNA-binding protein | PHAGE_Pseudo_PA10 | NC_041903 | 5.33E-94 |
| 45 | terminase small subunit | PHAGE_Pseudo_vB_PaeM_C2_10_Ab02 | NC_042113 | 4.61E-114 |
| 48 | portal protein | PHAGE_Pseudo_Zigelbrucke | NC_041904 | 6.87E-140 |
| 54 | flap endonuclease | PHAGE_Pseudo_PA10 | NC_041903 | 1.98E-27 |
| 71 | putative HNH homing endonuclease | PHAGE_Pseudo_Zigelbrucke | NC_041904 | 4.31E-24 |
| 72 | deoxynucleoside kinase | PHAGE_Pseudo_K5 | NC_030910 | 5.09E-66 |
| 74 | putative HNH homing endonuclease | PHAGE_Pseudo_phiMK | NC_031110 | 4.94E-67 |
| 80 | putative DNA polymerase | PHAGE_Pseudo_Zigelbrucke | NC_041904 | 5.15E-37 |
| 82 | deoxynucleoside kinase | PHAGE_Pseudo_phiMK | NC_031110 | 7.74E-50 |
| 85 | putative replicative DNA helicase | PHAGE_Pseudo_vB_PaeM_MAG1 | NC_031073 | 2.97E-27 |
| 87 | putative methyltransferase | PHAGE_Pseudo_Zigelbrucke | NC_041904 | 1.28E-101 |
| 90 | MazF family toxin-antitoxin system protein | PHAGE_Pseudo_Zigelbrucke | NC_041904 | 1.11E-38 |
| 100 | capsid decoration protein | PHAGE_Pseudo_phiMK | NC_031110 | 1.78E-45 |
| 103 | D11 protein | PHAGE_Pseudo_vB_PaeM_C2_10_Ab02 | NC_042113 | 2.27E-134 |
| 104 | DNA-binding protein | PHAGE_Pseudo_Zigelbrucke | NC_041904 | 2.35E-94 |
| 110 | TraG-like protein | PHAGE_Pseudo_K5 | NC_030910 | 3.09E-36 |
| 113 | membrane protein | PHAGE_Pseudo_K5 | NC_030910 | 2.72E-42 |
| 124 | DNA primase | PHAGE_Pseudo_K5 | NC_030910 | 9.54E-44 |
| 129 | major tail protein | PHAGE_Pseudo_Zigelbrucke | NC_041904 | 8.24E-115 |
| 130 | DNA helicase | PHAGE_Pseudo_PA10 | NC_041903 | 1.64E-40 |
| 132 | HNH endonuclease | PHAGE_Pseudo_PA10 | NC_041903 | 9.41E-42 |
| 135 | endolysin | PHAGE_Pseudo_phiMK | NC_031110 | 2.48E-111 |
| 136 | terminase large subunit | PHAGE_Pseudo_Zigelbrucke | NC_041904 | 1.20E-22 |
| 143 | tail fibers protein | PHAGE_Pseudo_vB_PaeM_C2_10_Ab02 | NC_042113 | 1.37E-19 |
| 148 | baseplate wedge | PHAGE_Pseudo_vB_PaeM_C2_10_Ab02 | NC_042113 | 4.47E-139 |
| 149 | tail fibers protein | PHAGE_Pseudo_Zigelbrucke | NC_041904 | 1.45E-52 |
| 160 | portal protein | PHAGE_Pseudo_vB_PaeM_G1 | NC_041968 | 2.25E-40 |
| 164 | tail fibers protein | PHAGE_Pseudo_phiMK | NC_031110 | 8.54E-37 |

**TABLE S1** Putative functional proteins of phages SPA01 and SPA05 (cont.)

| **Phage SPA05** | | | | |
| --- | --- | --- | --- | --- |
| **ORF no.** | **Putative function** | **Sequence similarity** | **GenBank ID** | **E-value** |
| 2 | tail assembly protein | PHAGE_Pseudo_Zigelbrucke | NC_041904 | 4.41E-17 |
| 3 | HNH endonuclease | PHAGE_Pseudo_Zigelbrucke | NC_041904 | 6.40E-72 |
| 4 | exonuclease | PHAGE_Pseudo_K5 | NC_030910 | 9.79E-110 |
| 5 | NrdH family redoxin | PHAGE_Pseudo_PA10 | NC_041903 | 2.94E-78 |
| 6 | HNH endonuclease | PHAGE_Pseudo_vB_PaeM_C2_10_Ab02 | NC_042113 | 1.57E-132 |
| 7 | nicotinamide mononucleotide transporter | PHAGE_Pseudo_PA10 | NC_041903 | 4.80E-29 |
| 10 | D14 protein | PHAGE_Pseudo_phiMK | NC_031110 | 1.38E-07 |
| 12 | putative phosphoesterase | PHAGE_Pseudo_Zigelbrucke | NC_041904 | 3.98E-66 |
| 17 | u-spanin | PHAGE_Pseudo_Zigelbrucke | NC_041904 | 3.84E-97 |
| 19 | holin | PHAGE_Pseudo_vB_PaeM_C2_10_Ab02 | NC_042113 | 7.29E-114 |
| 20 | endolysin | PHAGE_Pseudo_vB_PaeM_C2_10_Ab02 | NC_042113 | 4.13E-139 |
| 28 | prohead protease | PHAGE_Pseudo_phiMK | NC_031110 | 9.01E-75 |
| 49 | methyltransferase type 11 | PHAGE_Pseudo_Zigelbrucke | NC_041904 | 8.37E-30 |
| 50 | methyltransferase type 11 | PHAGE_Pseudo_vB_PaeM_MAG1 | NC_031073 | 1.34E-157 |
| 53 | putative phosphoesterase | PHAGE_Pseudo_vB_PaeM_LS1 | NC_048699 | 5.33E-33 |
| 54 | portal protein | PHAGE_Pseudo_vB_PaeM_G1 | NC_041968 | 2.25E-40 |
| 66 | tail fibers protein | PHAGE_Pseudo_Zigelbrucke | NC_041904 | 4.65E-55 |
| 67 | tail fibers protein | PHAGE_Pseudo_Zigelbrucke | NC_041904 | 1.21E-138 |
| 70 | tail fibers protein | PHAGE_Pseudo_vB_PaeM_C2_10_Ab02 | NC_042113 | 2.36E-44 |
| 73 | D5 protein | PHAGE_Pseudo_Zigelbrucke | NC_041904 | 2.05E-38 |
| 76 | minor tail protein | PHAGE_Pseudo_PA10 | NC_041903 | 1.33E-167 |
| 78 | tail tip protein | PHAGE_Pseudo_PA10 | NC_041903 | 1.16E-24 |
| 79 | prohead protease | PHAGE_Pseudo_K5 | NC_030910 | 1.39E-70 |
| 80 | tail fiber protein | PHAGE_Pseudo_PA10 | NC_041903 | 1.20E-22 |
| 84 | exonuclease | PHAGE_Pseudo_PA10 | NC_041903 | 1.84E-59 |
| 85 | endolysin | PHAGE_Pseudo_phiMK | NC_031110 | 1.36E-106 |
| 86 | prohead protease | PHAGE_Pseudo_Zigelbrucke | NC_041904 | 2.46E-35 |
| 88 | HNH endonuclease | PHAGE_Pseudo_PA10 | NC_041903 | 9.41E-42 |
| 89 | putative transcriptional regulator | PHAGE_Pseudo_PA10 | NC_041903 | 1.47E-52 |
| 90 | DNA helicase | PHAGE_Pseudo_PA10 | NC_041903 | 1.64E-40 |
| 91 | major tail protein | PHAGE_Pseudo_Zigelbrucke | NC_041904 | 1.65E-115 |
| 96 | D14 protein | PHAGE_Pseudo_K5 | NC_030910 | 2.77E-48 |
| 97 | DNA primase | PHAGE_Pseudo_K5 | NC_030910 | 6.84E-43 |
| 98 | flap endonuclease | PHAGE_Pseudo_K5 | NC_030910 | 3.67E-47 |
| 105 | membrane protein | PHAGE_Pseudo_K5 | NC_030910 | 8.05E-43 |
| 106 | DNA-binding protein | PHAGE_Pseudo_K5 | NC_030910 | 5.89E-75 |
| 114 | DNA-binding protein | PHAGE_Pseudo_Zigelbrucke | NC_041904 | 4.29E-94 |
| 119 | capsid decoration protein | PHAGE_Pseudo_phiMK | NC_031110 | 4.78E-46 |
| 121 | portal protein | PHAGE_Pseudo_vB_PaeM_MAG1 | NC_031073 | 3.91E-176 |
| 130 | putative DNA methylase | PHAGE_Pseudo_Zigelbrucke | NC_041904 | 2.84E-22 |
| 131 | putative methyltransferase | PHAGE_Pseudo_Zigelbrucke | NC_041904 | 8.27E-102 |
| 133 | putative replicative DNA helicase | PHAGE_Pseudo_vB_PaeM_MAG1 | NC_031073 | 4.32E-29 |

**TABLE S1** Putative functional proteins of phages SPA01 and SPA05 (cont.)

| **Phage SPA05** | | | | |
| --- | --- | --- | --- | --- |
| **ORF no.** | **Putative function** | **Sequence similarity** | **GenBank ID** | **E-value** |
| 136 | deoxynucleoside kinase | PHAGE_Pseudo_phiMK | NC_031110 | 1.01E-50 |
| 138 | putative DNA polymerase | PHAGE_Pseudo_Zigelbrucke | NC_041904 | 5.15E-37 |
| 144 | putative HNH homing endonuclease | PHAGE_Pseudo_phiMK | NC_031110 | 1.42E-55 |
| 146 | deoxynucleoside kinase | PHAGE_Pseudo_K5 | NC_030910 | 5.09E-66 |
| 147 | putative HNH homing endonuclease | PHAGE_Pseudo_Zigelbrucke | NC_041904 | 4.31E-24 |
| 162 | flap endonuclease | PHAGE_Pseudo_PA10 | NC_041903 | 1.98E-27 |
| 168 | portal protein | PHAGE_Pseudo_Zigelbrucke | NC_041904 | 4.84E-140 |
| 171 | terminase small subunit | PHAGE_Pseudo_vB_PaeM_C2_10_Ab02 | NC_042113 | 2.20E-113 |
| 173 | DNA-binding protein | PHAGE_Pseudo_PA10 | NC_041903 | 1.87E-94 |
| 175 | tail assembly protein | PHAGE_Pseudo_K5 | NC_030910 | 7.49E-111 |

| **Phage** | **Taxonomy**  **(Family, Genus)** | **Genome Size** | **GenBank** | **Recognized Bacterial Receptor** |
| --- | --- | --- | --- | --- |
| PhiKZ | *Myoviridae*, *Phikzvirus* | 280334 | NC_028999.1 | type IV pili |
| PhiPA3 | *Myoviridae*, *Phikzvirus* | 309208 | NC_028999.1 | Unknown |
| SPA01 | *Myoviridae*, *Pakpunavirus* | 93536 | NC_073602.1 | This study |
| SPA05 | *Myoviridae*, *Pakpunavirus* | 93656 | NC_073604.1 | This study |
| PAK_P1 | *Myoviridae*, *Pakpunavirus* | 93198 | NC_015294.2 | Lipopolysaccharide |
| K8 | *Myoviridae*, *Pakpunavirus* | 93879 | NC_028817.1 | Lipopolysaccharide |
| PaP1 | *Myoviridae*, *Pakpunavirus* | 91715 | NC_019913.1 | Lipopolysaccharide |
| JG004 | *Myoviridae*, *Pakpunavirus* | 93017 | NC_019450.1 | Lipopolysaccharide |
| vB_PaeM_MAG1 | *Myoviridae*, *Pakpunavirus* | 94555 | NC_031073.1 | Unknown |
| PAK_P2 | *Myoviridae*, *Pakpunavirus* | 92495 | NC_022967.1 | Lipopolysaccharide |
| vB_PaeM_C2−10_Ab1 | *Myoviridae*, *Pakpunavirus* | 92777 | NC_019918.1 | Unknown |

**TABLE S2** Recognized Bacterial Receptor of phages used in this study and other closely related phages.


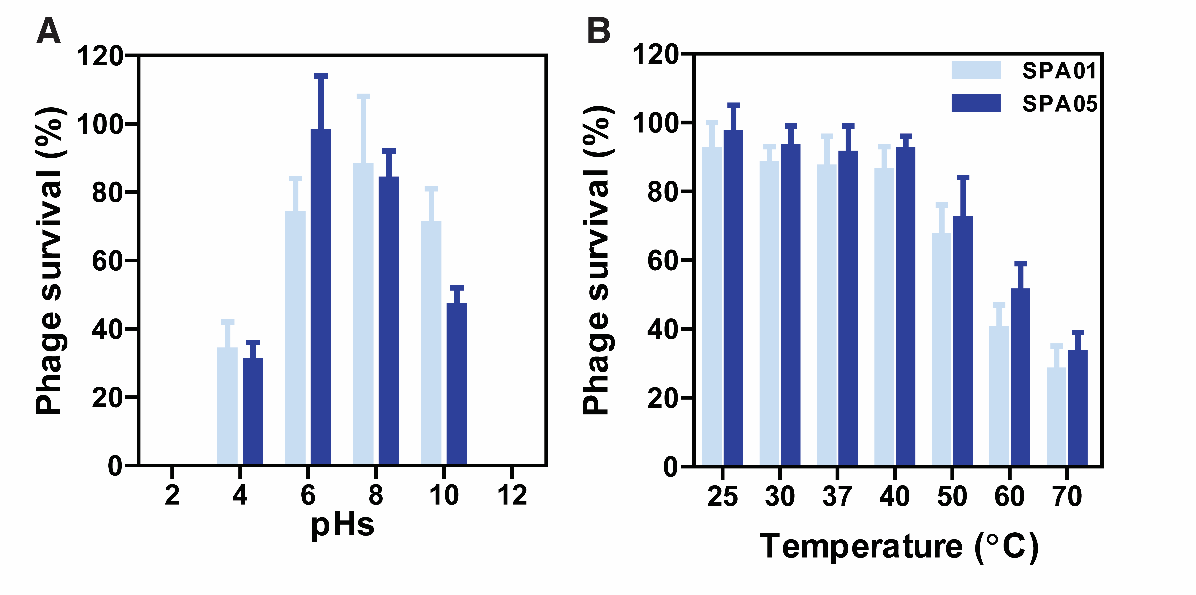


**FIG S1** Thermal and pH stability of phages SPA01 and SPA05. The activity of phages SPA01 and SPA05 at different pH levels **(A)**. The activity of phages SPA01 and SPA05 at different temperatures **(B)**. Each experiment was performed in triplicates and, means and standard deviations are indicated.


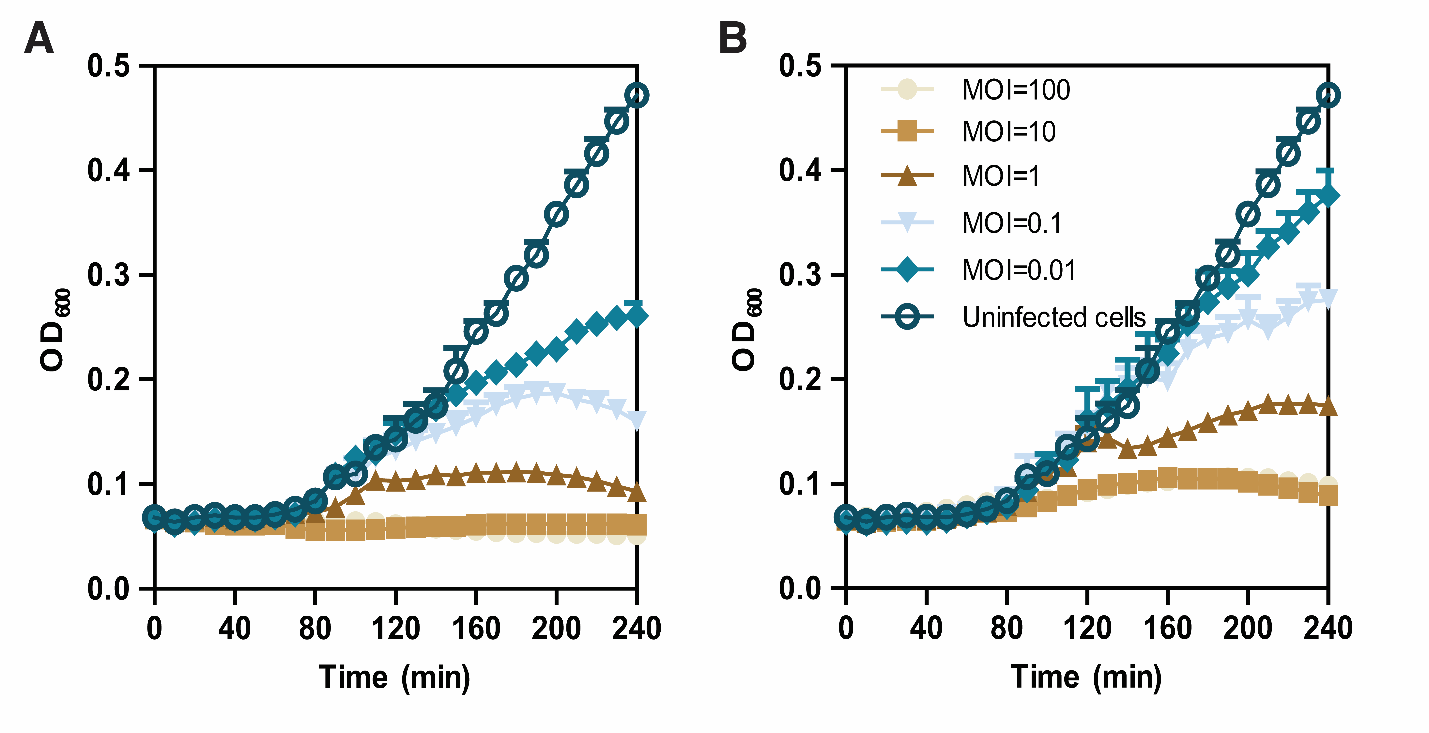


**FIG S2** Planktonic cell lysis kinetics of PhiKZ **(A)** and PhiPA3 **(B)** at MOIs of 0.01, 0.1, 1, 10, and 100 against *P. aeruginosa* PAO1. Each experiment was performed in triplicates and, means and standard deviations are indicated.


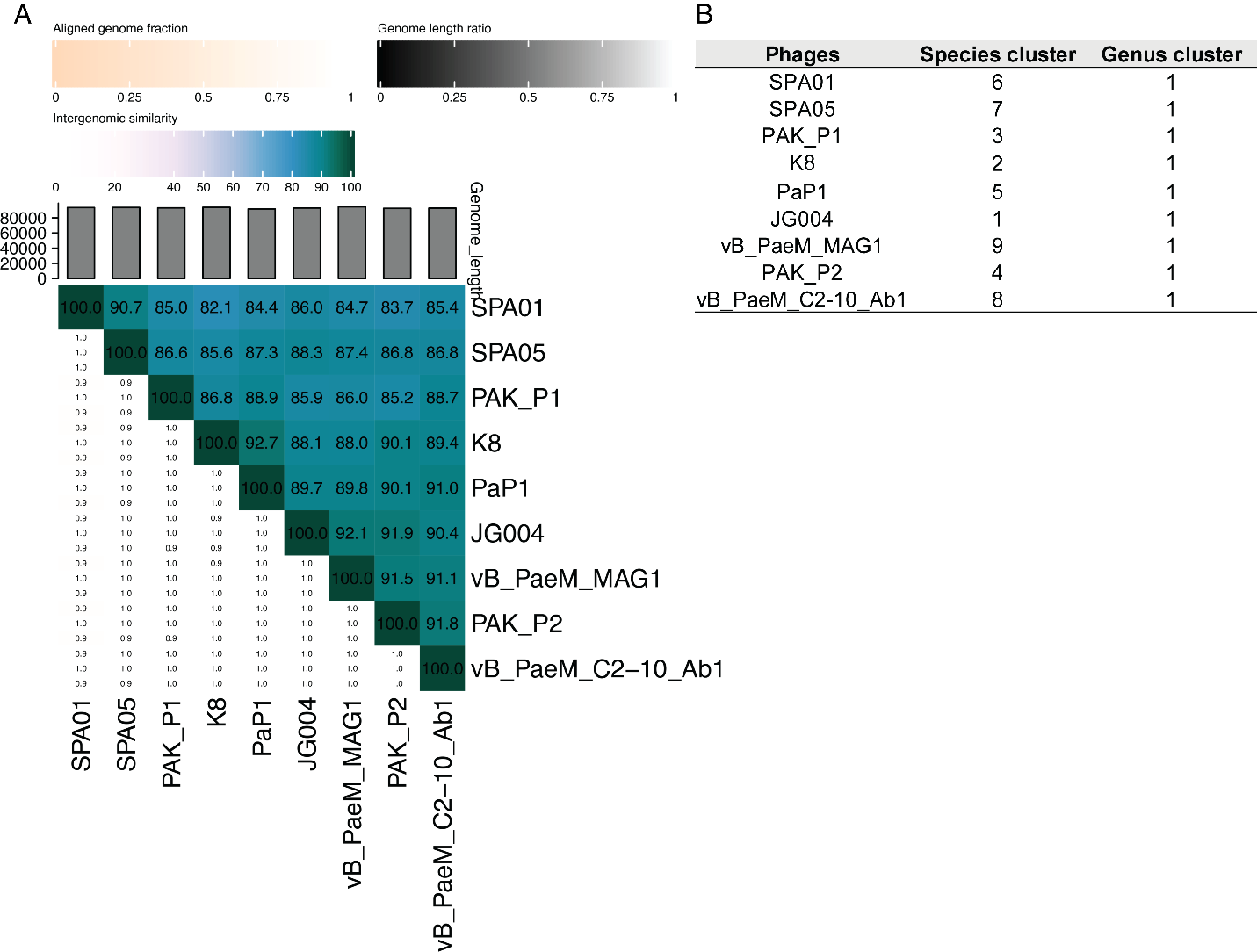


**FIG S3** VIRIDIC heatmap of the 9 *Pakpunavirus*. Intergenomic similarities of the *Pakpunavirus* are shown on the right side with the percent similarity and colored scale **(A)**. Species and genus clusters of *Pakpunavirus* are shown in **(B)**.
